# Hairy surfaces by cold drawing leading to dense lawns of high aspect ratio hairs

**DOI:** 10.1038/s41598-022-13419-3

**Published:** 2022-06-15

**Authors:** Stefan Müllers, Mara Florea-Hüring, Bernhard von Vacano, Bernd Bruchmann, Jürgen Rühe

**Affiliations:** 1 Department of Microsystems Engineering—IMTEK, University of Freiburg, Georges-Köhler-Allee 103, 79110 Freiburg, Germany; 2 BASF SE, Advanced Materials and Systems Research, Carl-Bosch-Strasse 38, 67056 Ludwigshafen, Germany; 3 livMatS@Freiburg Institute for Interactive Materials and Bioinspired Technologies (FIT), University of Freiburg, Georges-Köhler-Allee 105, 79110 Freiburg, Germany

**Keywords:** Engineering, Materials science, Nanoscience and technology

## Abstract

The surfaces of many organisms are covered with hairs, which are essential for their survival in a complex environment. The generation of artificial hairy surfaces from polymer materials has proven to be challenging as it requires the generation of structures with very high aspect ratios (AR). We report on a technique for the fabrication of surfaces covered with dense layers of very high AR nanoscale polymer hairs. To this, templates having pores with diameters of several hundred nanometers are filled with a polymer melt by capillary action. The polymer is then allowed to cool and the template is mechanically removed. Depending on the conditions employed, the formed structures can be a simple replica of the pore, or the polymer is deformed very strongly by cold drawing to yield in long hairs, with hair densities significantly up to 6,6 × 10^8^ hairs/cm^2^ at AR of much higher than 200. The mechanism of hair formation is attributed to a delicate balance between the adhesion forces of the polymer in the pore and the yield force acting on it during mechanically demolding. We demonstrate how with very little effort and within a timescale of seconds unique topographies can be obtained, which can dramatically tailor the wetting properties of common polymers.

## Introduction

The surfaces of many animals (or in some cases also plants) are at least partially covered with dense layers of hairs^1,2^. Hairs can serve many purposes: They can protect against the impact of UV or infrared radiation^3^, or they can shield against direct water contact of the body during rain exposure^4,5^ or during swimming^6,7^. Hairs can also have a thermoregulation function to stabilize the body temperature^2^. To do so, they act as an insulating layer^6^, which reduces heat uptake or heat loss, as they entrap significant amounts of air. Alternatively, by taking up and distributing sweat over a larger surface area hairs can increase the rate of water evaporation and thus cool down the body they are covering. In some cases they also play a role in social interactions by contributing to the distribution of odors, such as pheromons^1^.

Biological hairs are essentially high aspect ratio, keratin-based materials^1^, firmly anchored to the surface of the skin they cover. They form a dense “lawn” in which the interhair distance is significantly smaller than the length of an individual hair^8^. An aspect ratio, AR > 100 is characteristic for natural hairy plant surfaces, such as for example the lady’s mantle leaves^9,10^. For human hair aspect ratios of even higher than 1000 can be observed.

In the technological field, high AR structures can be written into rather rigid materials, mostly silicon, through photo-, x-ray or electron beam lithography followed by highly specific etching processes^8,11,12^. An example of a system where very high AR structures were generated for an interesting application, is the work of Chang and Sakdinawat^13^. They employ electron beam lithography and a metal-assisted chemical etching technique for the fabrication of ultra-high AR (> 120), high-resolution nanofeatures, which can be used for generating an imaging optics for hard X-rays. However, in contrast to the large number of publications on high AR micro and nanostructured silicon surfaces, much less has been reported when polymeric materials are considered^14,15^. Key methods for the generation of polymer micro and nanostructures are photolithography and microreplication, the latter approach is especially preferred when fabrication of structured areas on a large scale is desired^12,16^.

Polymer nano- or microhairs can be generated using replica-molding techniques in which porous templates are employed^10,17,18^. Several examples, in fact, made use of natural surface masters, such as hairy leaves^10,19^ or actual insects^17^ for obtaining negative molds, which in turn led to identical polymer replica morphologies. Reproducing natural hairy surfaces with very high AR (> 100) remains, however, a challenge since especially demolding of such high AR structures from the template is rather difficult. Recently, using a natural bovine dental template, Tiller and coworkers have successfully generated ultra-long acrylate resin filaments possessing an AR of up to 200^9^. The template-assisted polymerization resulted in surfaces mimicking the topography of a *Corokia cotoneaster* leaf. However, as natural templates are typically rather small in size and they are dissolved or etched away in such approaches, the sample size, obtained through such pathways are intrinsically limited and large scale substrates cannot be modified.

Synthetic porous templates, such as commercial polymeric membranes, have also been combined with polymer films for generating hairy surfaces. An interesting approach in this direction represents the work of Fearing and Sigmund, where hairy polypropylene (PP) surfaces were obtained via a microreplication technique which uses a polycarbonate (PC) membrane as a template^20,30^. The membrane and the substrate to be modified, a PP foil, were pressed between two glass slides under vacuum, followed by manual separation. This way water-repellent surfaces decorated with microhairs resembling those found in arthropods were obtained. The hairs had diameters closely resembling the pores and lengths up to 10 microns. Although the approach provides a simple route to mimicking natural surfaces, the mechanism of hair formation has not been reported yet. Another example reports on a synthetic gecko adhesive based on PP fibrillar arrays^18^. The tubular structures displaying an AR of up to 30 have been generated by etching the PC membrane upon casting.

Nanodrawing is another strategy for generating high AR polymer structures, which relies on a strong adhesion between the mold and the polymer^21–24^. For instance, elongated hierarchical polymeric nanohairs (AR ≈ 10) mimicking the gecko foot hairs were obtained using a multi-branched anodic aluminum oxide (AAO) template^24^. The work of adhesion plays a critical role in nanodrawing, as reported in detail by Suh and coworkers^21,22^. An interesting three component system was described, comprising of a solid substrate, a poly(urethane acrylate) PUA mold, and a spin coated layer of the polymer film. By carefully controlling the capillarity process and the adhesive force at the mold/polymer and polymer/substrate, respectively, elongated PS and PMMA nanohairs (AR > 20) were successfully generated over a large area.

## Results

The formation of surfaces covered with dense “lawns” of polymer hairs can be achieved using a rather simple and very fast process. To this end, the polymer substrate to be covered with the hairs is heated, so that a thin surface layer becomes slightly molten and is then brought into contact with a porous substrate, i.e., an ion track etched membrane. Due to capillary action the pores are at least partially filled with the polymer melt and subsequently the system is left to cool down to room temperature. Upon separation of polymer substrate and membrane, the some part of the polymer sticking in the pores of the membrane is slowly pulled out and by this process becomes strongly elongated while the majority of the is held back in the micropore by frictional forces. We study the mechanism of hair formation and the influence of the process parameters onto the “hair style” of the thus obtained hairy surface.

**Figure 1:**
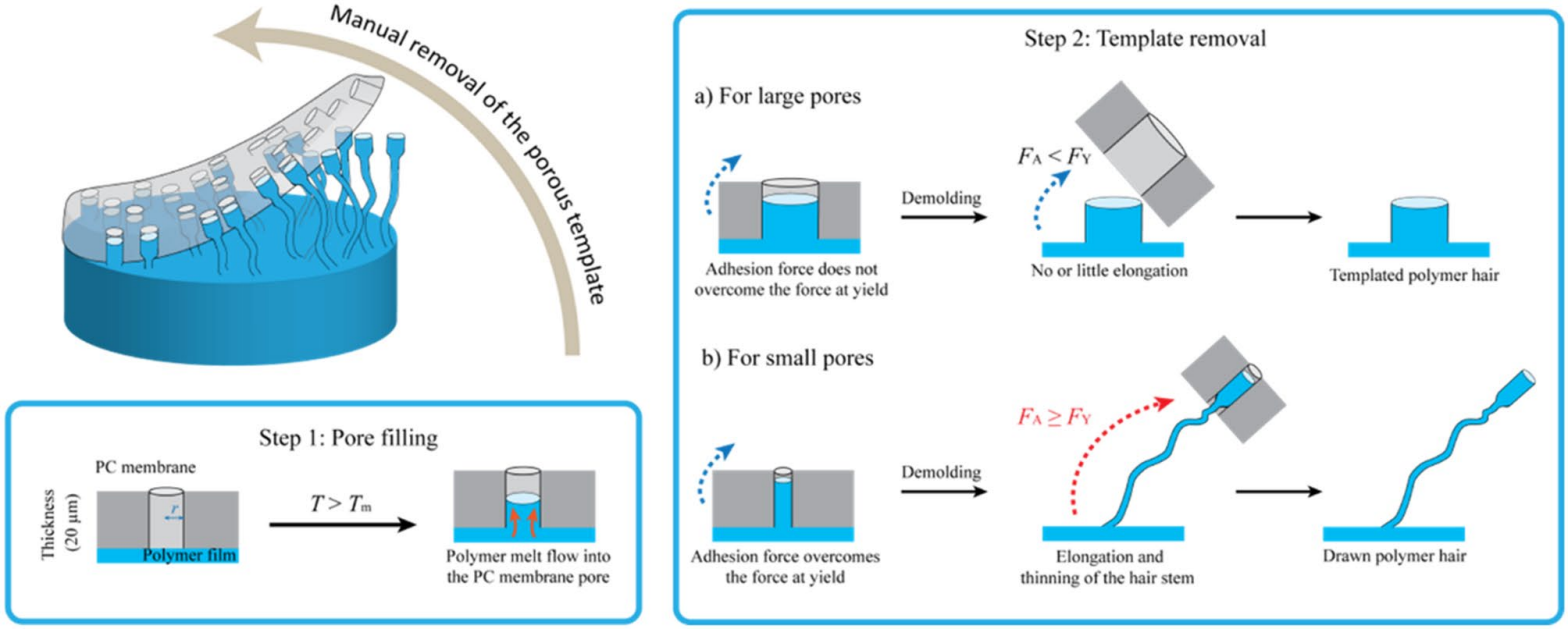
Pore-assisted modifications of polymer film surfaces using ion-track etched PC membranes as porous templates. Depending on the pore size either (**a**) microreplicated cylinders or (**b**) drawn hair-like nano- and microstructures can be generated.

High-density polyethylene, HDPE, films are heated to 170 °C by placing them on a hot plate, slightly above the melting point of the polymer (*T*_m_ ≈ 134 °C). These films are then brought into contact with commercially available, ion-track etched PC membranes (Fig. 1, top). Within seconds, the polymer melt fills the pores of the PC template due to capillary action. Ion track etched membranes contain circular pores which are open on both ends, which is very important as air contained in the pore is easily released during filling. After cooling down of the substrate, mechanical (e.g. manual or machine controlled) removal of the template from the polymer film results in either regular microreplicated cylinders or conversely, in partly entangled, but mostly freestanding, strongly elongated polymer hairs (Fig. 1). The creation of hairs in the case where the separation of membrane and substrate is machine controlled, is highly reproducible with homogenously covered surfaces.

**Figure 2:**
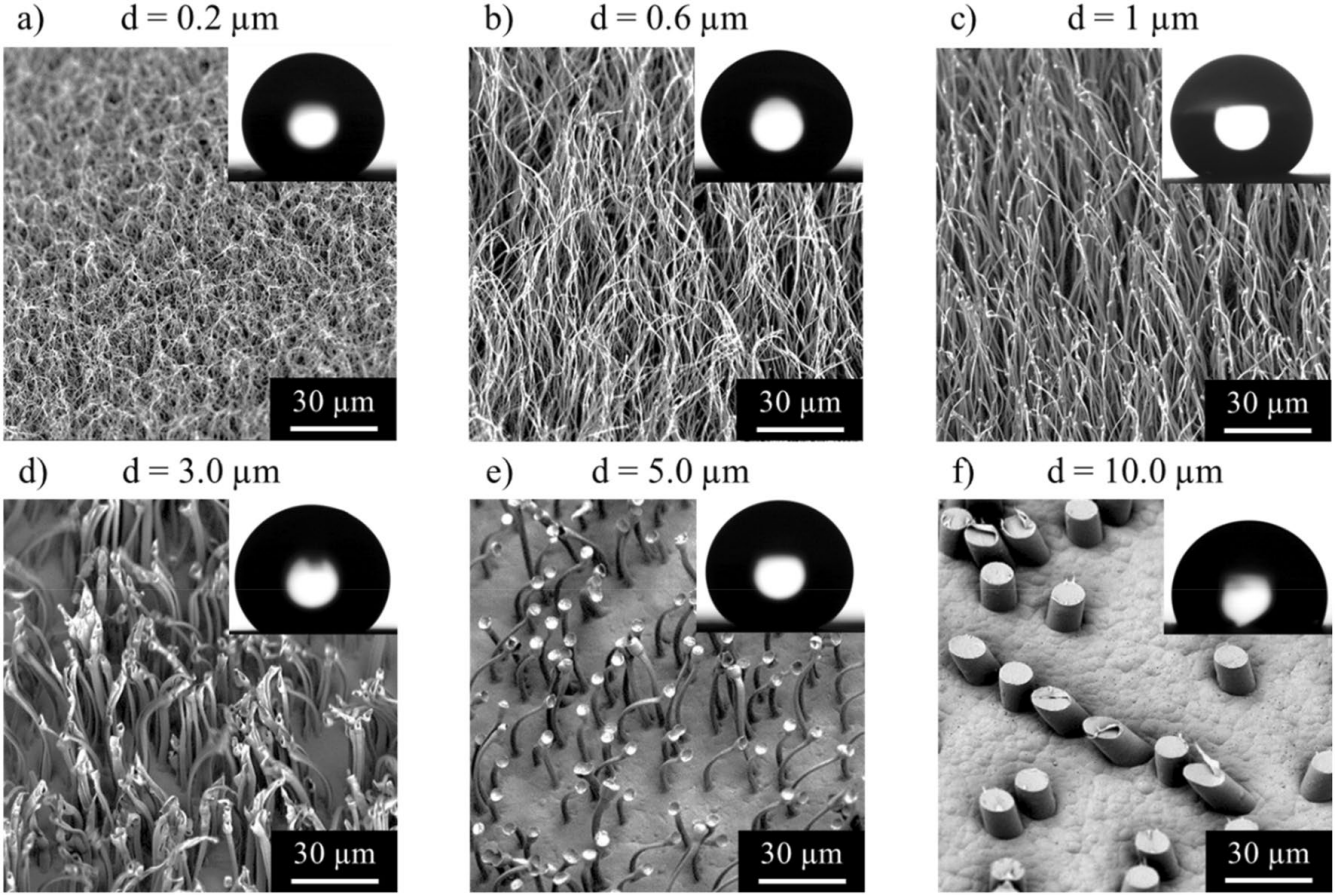
Scanning electron micrographs corresponding to various HDPE nano- and microstructures obtained by using PC membranes with pore diameter ranging from 0.2 µm to 10.0 µm. All samples were obtained under identical fabrication parameters (600 s seconds at 170 °C). The structures (**a–d**) show superhydrophobic behavior (*θ* = 160° ± 10°; *θ*_roll-off_ < 10°).

The pore size is one of the critical parameters for the various surface topographies obtained. When template membranes with large pores i.e., *d* ≥ 10 μm (Fig. 2f) are employed, a rather classical replication process occurs, which results in the formation of HDPE cylinders, whose dimensions and aspect are dictated by the pores of the template employed. As the SEM micrographs in Fig. 2a–e show, the use of membranes with smaller pores, i.e., *d* ≤ 5 μm resulted in highly disordered, hair-like surfaces. The length of these hairs was several times larger than the membrane thickness employed. The drawing of the polymer nano- and microstructures during demolding causes them to simultaneously elongate, while reducing their diameter. In some cases the tips of the resulting hairs carry a bulge, whose cross-section dimensions are reminiscent of the template pore (Fig. 1b). When membranes with pore diameters in the range of 5 µm are used, a transition regime is observed, where microreplicated and elongated structures are simultaneously generated (Fig. 2e). Table 1 compiles the aspect characteristics of HDPE microstructures generated from templates having different pore sizes as well as their newly emerging wetting properties. When membranes with pore diameters of 200 or 600 nm were used hairs with aspect ratios of 200 were obtained. At very small pore sizes (d = 0,2 µm) the AR were indeed a little smaller than those at d = 0,6 µm as hair rupture occurred during demolding. Even higher aspect ratios > 200 and occasionally even over 400 have been observed at intermediate pore sizes, however, under such conditions the process is in a critical regime. Although qualitatively excellent reproducibility is observed, even small process variations lead to structural variations and the exact values of the AR are difficult to reproduce.

**Table 1:** Aspect details of the various ion-track etched polycarbonate membranes employed as templates as well as of the resulting HDPE microstructured films and their corresponding wetting behavior.

Pore diameter (μm)	Microstructure diameter (μm)	Microstructure height (μm)	Aspect ratio (AR)	Wetting, contact angle	
				Static CA (°)	Hysteresis (°)
−	None^a^	None^a^	−	94	30
0.2	0.1	20^b^	200	156	< 10
0.6	0.3	80^b^	> 200	154	< 10
1.0	0.5	70	140	151	< 10
3.0	1.5	45	30	151	< 10
5.0	2.5	35	14	157	< 10
10.0	10.0	12	1.2	120	50

^a^HDPE film as received. ^b^approximated value.

In order to understand the different structure formation regimes observed, we examined the relevant forces at play during the fabrication process. To this end, we decided to simplify the description of the system by examining the formation of one single hair. This seems justified as each pore is filled individually and also the elongation of each formed post happens without the influence from his neighbours. Additionally, due to the ion track method employed for pore generation the pores have an almost perfect cylindrical shape and are very uniform in size (dispersity < 10%) and pore to pore variations are rather small. The process of hair formation consists of two well-defined stages (Fig. 1): filling of the pore with polymer melt via capillary forces, which occurs very rapidly, followed by template removal upon cooling and mechanically drawing the polymer in the pore through cold flow and eventually separation of polymer structure and pore (demolding). In order to stretch the hair, the interfacial adhesion between the HDPE and the PC pore walls must be higher than the drawing force. For separation of polymer and pore, i.e. demolding, the interfacial adhesion must be overcome in the drawing process. Interestingly, a strong adhesive strength, between σ_t_ = 3 MPa (own measurement) and σ_t_  = 30 MPa^25^ has been reported for polyethylene/polycarbonate surfaces. Combining the adhesive strength between the two polymers, σ_t_, and the relevant area of contact, i.e., the surface area of the pore side walls (2π*rh*), leads to an adhesion force, *F*_A_ (Eq. 1**)**, where *r* is the pore radius and *h* denotes the height of the polymer column in the pore of the PC membrane.

1 $${F}_{A}={\sigma }_{i}\times 2\pi rh$$

Next to the radius of the pore, the height of the filling of the pore is a critical parameter. An estimation of the degree of pore filling at different contact times, i.e., in situations, when the system is still in the process of pore filling, can be obtained by the Washburn equation (Eq. 2)^24–26^, in which *t* represents the time (in seconds) required for a liquid having a dynamic viscosity η and surface tension γ (here 26.5 mN/m^26,27^) to penetrate a distance *h* into a pore with a radius *r.*

2 $${h}^{2}= \frac{\gamma \times r\times \mathrm{cos}(\theta )}{2\eta }t$$

**Figure 3:**
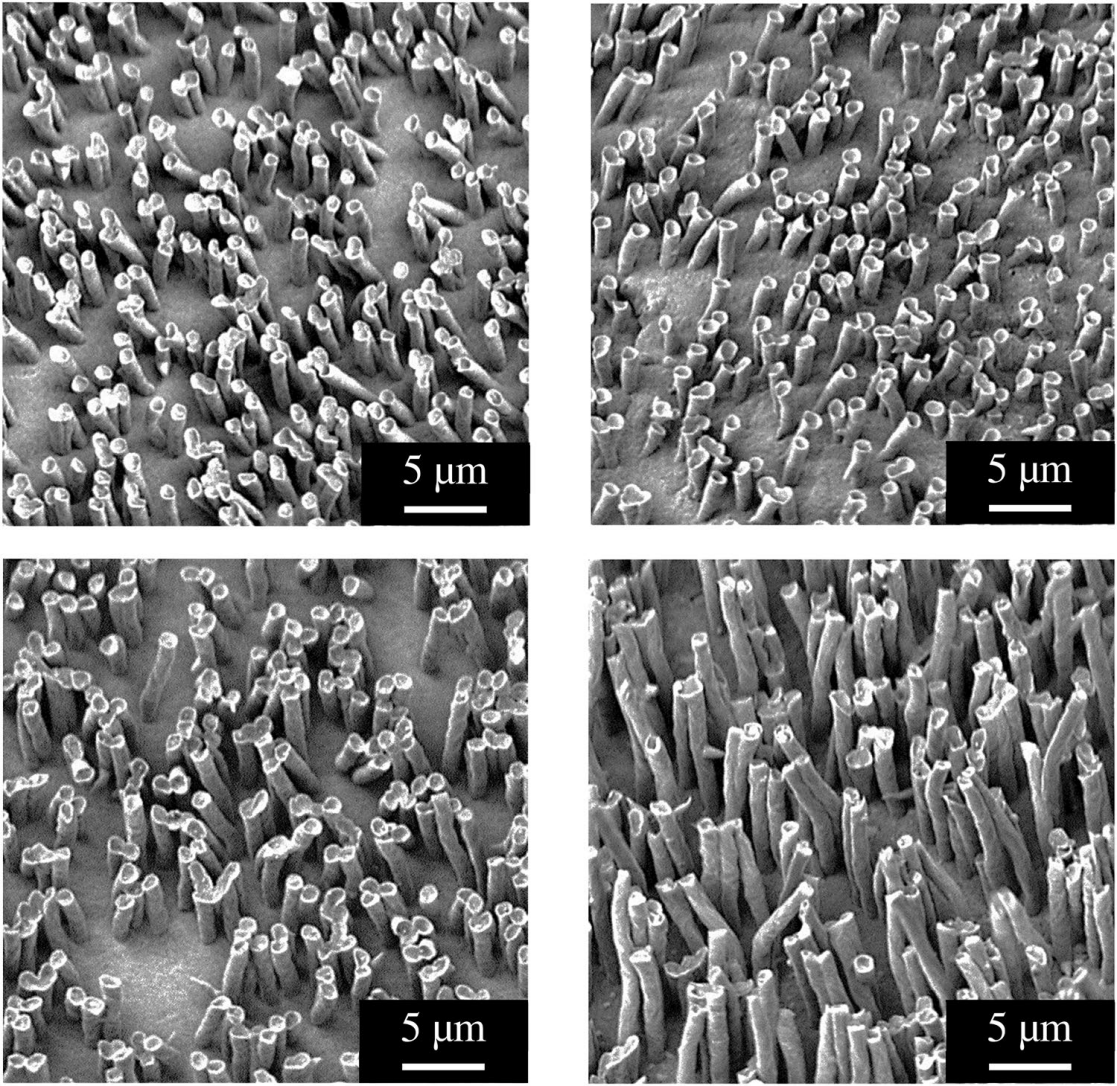
SEM images of HDPE structures after filling of the pores by capillary action (T: 170 °C), cooling down and dissolution of the PC membrane in dichloromethane (chemical demolding); contact times: (**a**) 30 s, (**b**) 60 s, (**c**) 300 s and (**d**) 600 s.

To this we have recorded the rheological properties of the employed HDPE at 150 °C. While the exact value of the shear rate inside of the pore could not be measured directly, from the filling times and flow speed (~ 0.1 μm/s) the shear rate is calculated to 0.2 s^−1^. At such a shear rate the polymer viscosity has a value of η = 3500 Pa•s. The contact angle of the planar PE substrate is $\theta =85^\circ$. The thus calculated capillary heights of the different pore sizes for a contact time of 30 s were found to gradually increase with the pore diameter from *h* = 1.0 μm for *d* = 0.2 μm to *h* = 6.9 μm for *d* = 10.0 μm from about 3 to 9 µm. The thus estimated values nicely correlate with the capillary heights obtained from electron microscopy investigations shown in Fig. 2. In these experiments the membrane was removed from the microstructured films by dissolving it in dichloromethane (‘chemical demolding’), which is a good solvent for polycarbonate-based materials (Fig. 3)^18^.

**Figure 4:**
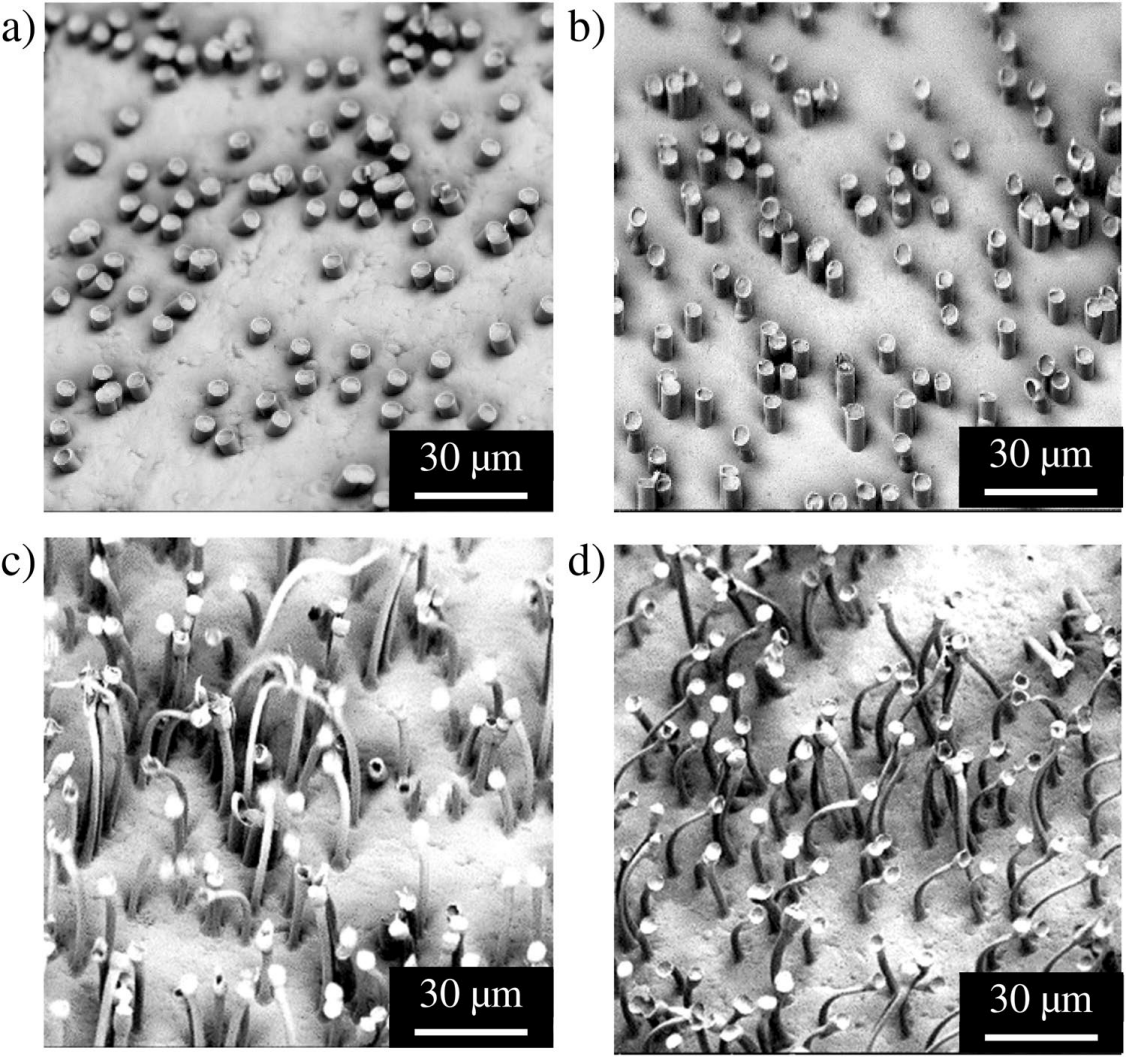
SEM images of a HDPE sample structured through contact with membranes with a pore diameter of 5 µm; contact temperature: T = 140 °C; contact times: contact times: (**a**) 30 s, (**b**) 60 s, (**c**) 300 s and (**d**) 600 s.

The importance of the filling height of the polymer in the pore onto the drawing process is exemplarily shown in Fig. 4. Here, essentially a series of identical samples are prepared with the only difference that the contact time and accordingly the filling height of the pores was different. It can be clearly seen that short contact times lead to practically no drawing, while at higher pore filling strongly elongated structures are obtained.

This is, by the way, also the reason while similar processes with open and closed pore substrates can give very different results. An important feature of the ion track etched membranes is that they have a continuous, open pore structure and do not contain blind holes, so that upon contact with the polymer melt and the capillary filling no air can become trapped in the structures. In case of blind holes the pressure build-up counteracts the filling of the pores and the amount of polymer inside the cavities depends on the balance between size of the pore and any added/reduced pressure. Theoretically, if the polymer would flow into the pore only on one side and fill it from the top the air could escape. This, however, is a rather delicate situation as easily air can become trapped due to small process variations. We tried membranes with closed pores and obtained not very reproducible results and only much lower aspect ratio hairs were obtained. Alternatively one could perform vacuum molding processes, which is technically not so easy to perform.

**Figure 5:**
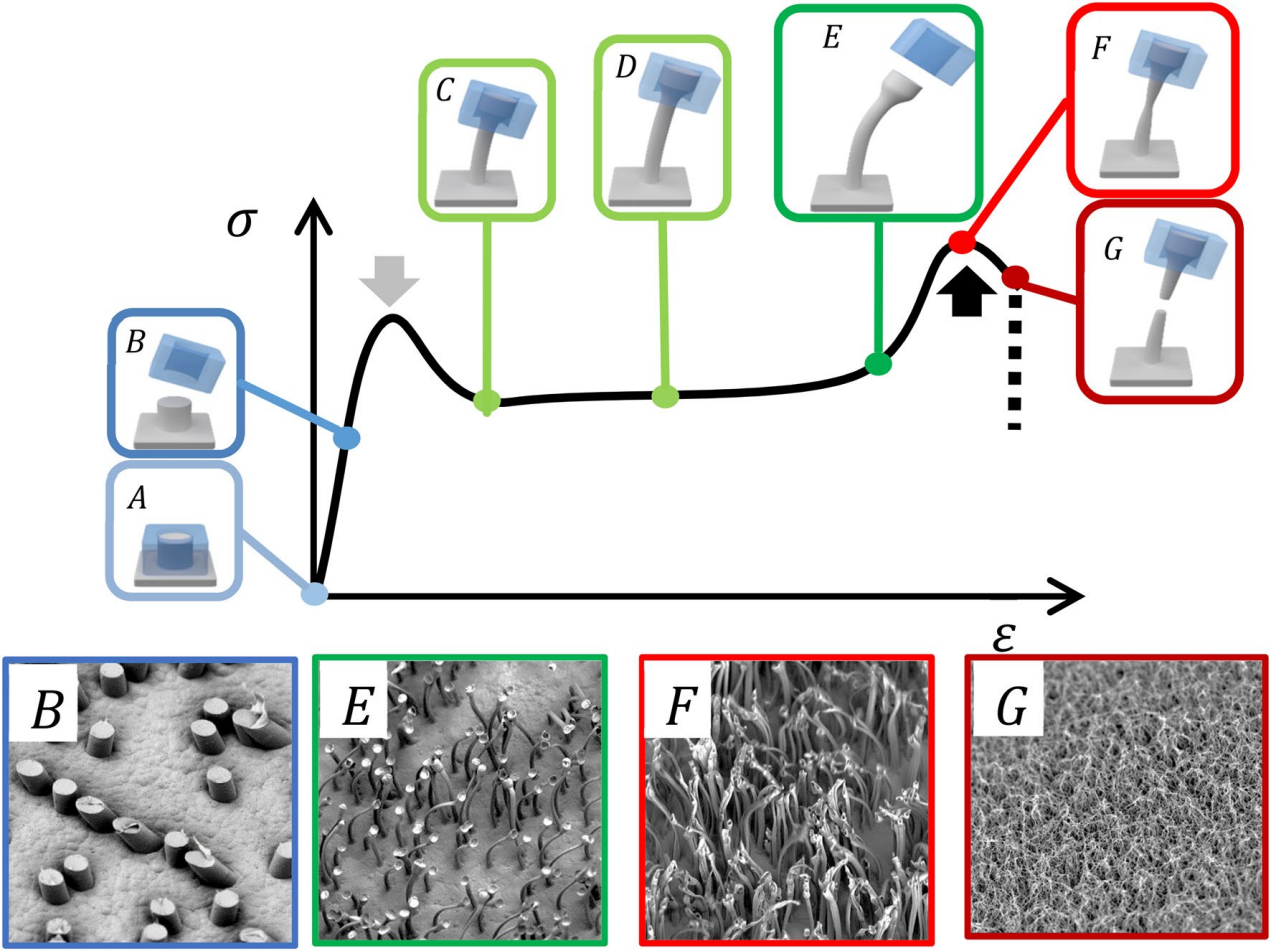
Schematic depiction of a stress strain-curve of a thermoplastic polymer and the hair formation process together with the corresponding SEM images of samples prepared according to scenarios (**B**,**E**,**F**,**G**) shown in the schematic; the grey arrow indicates the yield point, the black arrow the break point at which hair rupture occurs; (**A**) filling of the pore, (**B**) demolding with templating, (**C–E**) hair formation through cold drawing, (**F**,**G**) hair rupture.

The other significant force component expected to influence the topography of the final surface pattern is the yield force, *F*_Y_. According to a typical stress–strain curve (Fig. 5), a thermoplastic polymer will plastically deform when the applied stress surpasses the yield point. SEM images of structures obtained in scenarios B;E, F and G are shown in Fig. 4b. When the yield point is not surpassed, no elongation is observed and a templated structure is obtained. Above the yield point the polymer in the pores becomes elongated until the remaining polymer “plops out” and forms a cork-link bulge at the end of the hair. When scenarios E,F and G are compared the hair length is increased. At the same time the length of the “cork” at the end of the hair becomes smaller from E to F and is no longer visible in scenario G.

The tensile stress *F*_Y_ originating from manually peeling off the PC membrane acts transversally onto the cross section area (π*r*^2^) of the newly formed microstructure and on the yield stress ${\sigma }_{y}$ of the polymer (Eq. 3):

3 $${F}_{Y}={\sigma }_{y}\times \pi {r}^{2}$$

**Figure 6:**
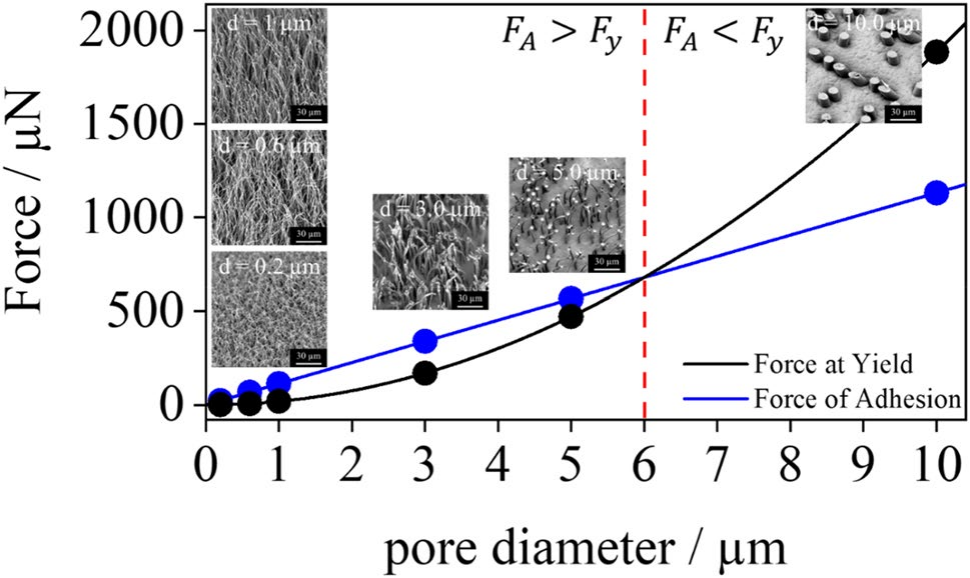
Forces of adhesion (black dots) and forces at yield (red dots) in dependence of the pore diameter employed. Note that the system was simplified to one pore and assumed to be under thermodynamic equilibrium, i.e., pores entirely filled. The intersection of the two force curves defines the two working regimes observed experimentally: cold drawing for the smaller pore diameter (*d* ≤ 5 μm), where *F*_A_ ≥ *F*_Y_, and microreplication for the larger pore diameter (*d* = 10 μm), where *F*_A_ < *F*_Y._

**Figure 7:**
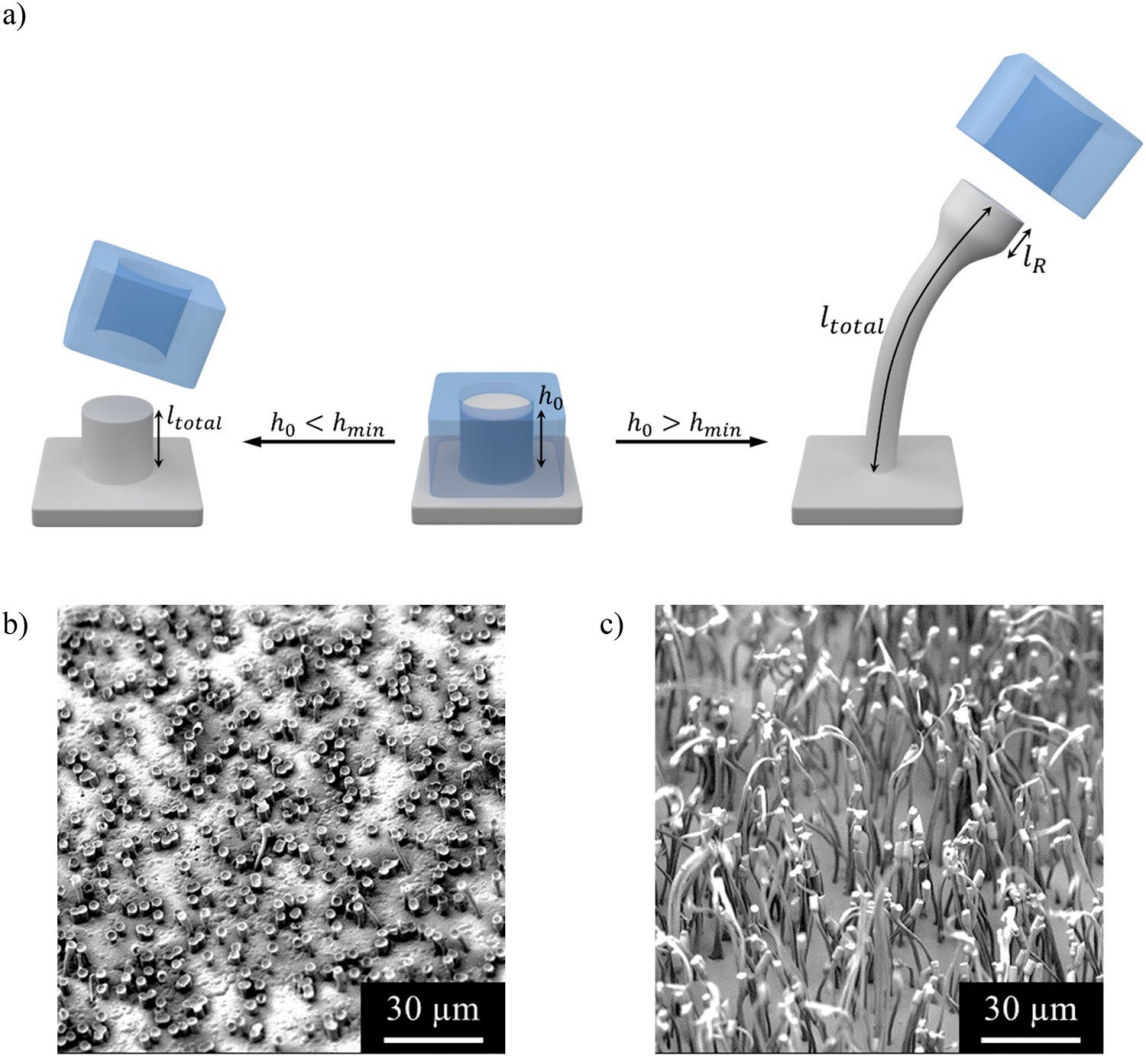
(**a**) Contact time dependence of the draw ratio. Depending on the filling level ℎ_0_ simple replication of the pores takes place or the process of cold stretching to hairy structures. SEM image of a patterned HDPE sample with PC-3 μm at (**b**) T = 150 °C , t = 300 s , no stretching takes place due to insufficient rise height, ℎ_0_ < ℎ_*min*_. (**c**) T = 150 °C, t = 600 s, the rise height increased by longer contact time which leads to successful stretching of the structures, ℎ_0_ > ℎ_*min*_.

For the HDPE used here, stress–strain experiments gave values of ${{\varvec{\sigma}}}_{{\varvec{y}}}=$ 24 MPa. Plotting the two forces as a function of the pore radius (assuming almost fully filled pores for all diameters) allows for a surprisingly clear distinction of two regimes (Fig. 6). In the small pore regime with *d* ≤ 5 μm the frictional force due to adhesion between the polymer filling the pore and the pore wall is higher than the force at yield, so that the polymer is firmly held inside of the pore. If now a sufficiently strong force is applied, the polymer will undergo cold drawing, resulting in long, elongated polymer hairs. However, as the yield force increases quadratically with the pore size, beyond a critical value, i.e., for *d* > 5 μm, the pull-out force surpasses the adhesion force leading to a classical microreplication process. In this case well defined polymer cylinders are formed, which have more or less the same dimensions as the template pores. At the border between the two regimes (i.e. at *d * ≥ 5 μm) the difference between the force of adhesion and the yield is very small, so that both the formation of short hairs and templating occur on the same substrate, depending of the height of filling. As shown in Figs. 2 and 5, this analysis correlates in an excellent manner with the experimental observations described above. However, as already briefly discussed above this balance between the two forces is not only related to the pore diameter but also a function of the height of filling. The filling needs to extend a certain level to offer enough contact area between polymer and template to lead to the minimum level of adhesion to initiate the elongation process (Fig. 7).

**Figure 8:**
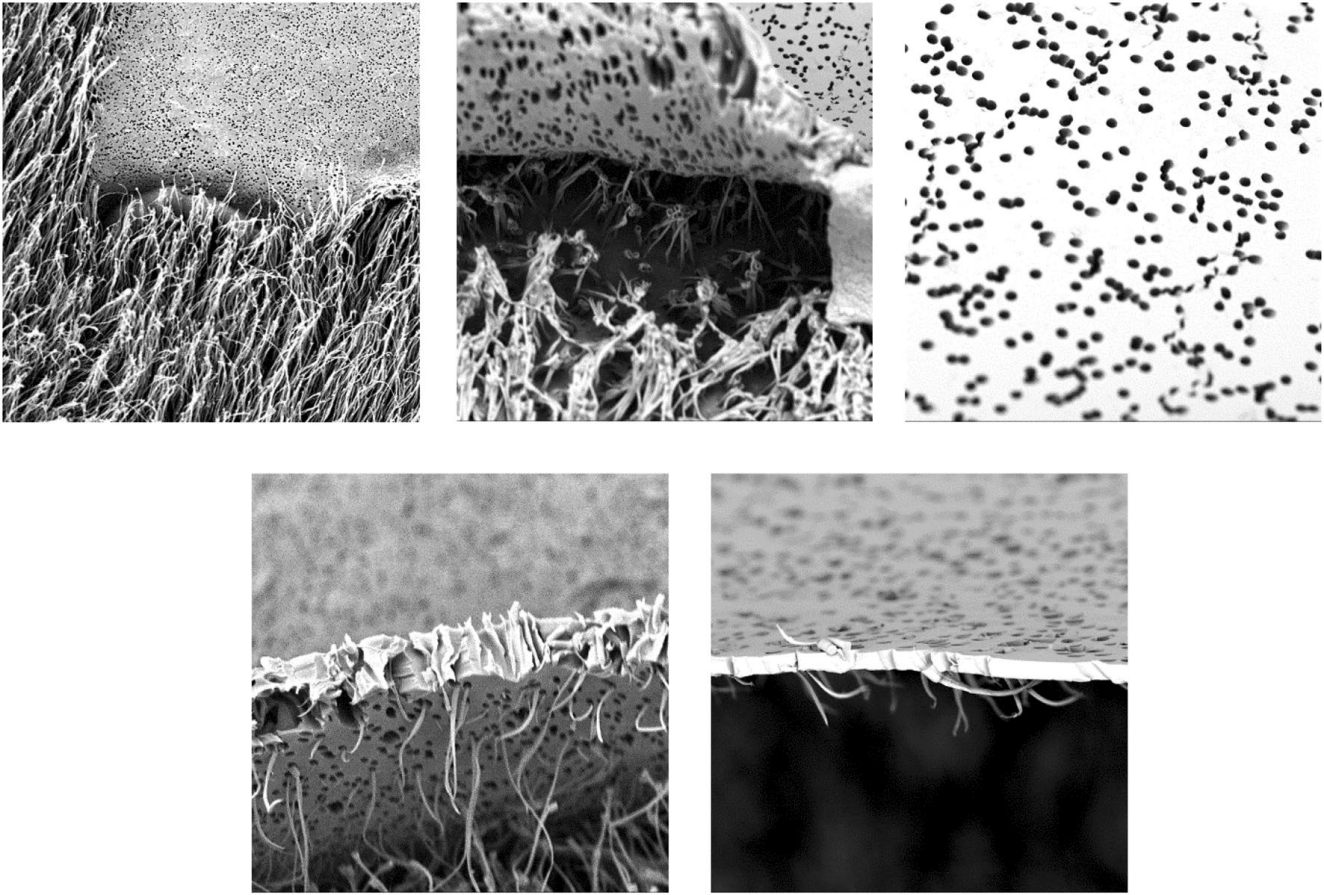
Reusability of the membranes after hair formation; upper row: after demolding under appropriate conditions (hair diameter close to 1 µm, room temperature demolding) the membranes can be separated easily from the hairy structures, no hairs remain in the pores; the membranes have an appearance as new and can be reused; lower row: for very small pores and high degrees or pore filling, i.${\varvec{\theta}}$ e. when the strain is higher than a critical value some of the hairs rupture and remain stuck in the membrane; the membrane can be reused only after cleaning.

Usually the membranes separate in a very clean way from the substrate (Fig. 8, upper row. However, it should be noted, that in some cases, when very small pores (*d* ≤ 0,6 μm) are used and the pore filling is very high, the forces occasionally exceed the break point of the polymer (situation G in Fig. 5). As a consequence of this some of the polymer hair ruptures during drawing while the hair density remains more or less the same. When the template membranes of such samples were inspected after demolding some broken off parts of the hairs were observed (Fig. 8 lower row).

It is interesting to note that as the material is drawn out of the pore (if d ≥ 0,6 µm), the filling is reduced so that the contact area between pore and polymer becomes smaller and the frictional force becomes gradually smaller. When now a lower critical friction value is reached, the residual polymer simply slides out without further elongation like a cork out of a bottle. This leads to the bulges at the end of the hairs visible in some of the micrographs, e.g. Fig. 2.

It is interesting to compare the process described here with an at first sight somewhat similar process described by Jeong et al.^22^ who used also membranes for cold drawing. However, (apart from the situation of open vs. pores closed on one side as discussed above) in the literature procedure for hair generation an experimental setup composed of a tri-component system (substrate, polymer film, mold) was used. In contrast we use here a two-component system i.e.. polymer/mold. This might seem a minor detail, but it renders the physics of the process very different. In the trilayer system the relation between the adhesion at the interfaces between polymer/mold vs. polymer/substrate is the determining factor for drawing vs. microreplication^22^, and determines in the end the aspect ratio of the structures obtained. In our bilayer system, the relation between friction/ adhesion and deformation force dominates. This is probably the reason why very different aspect ratios were obtained even though porous membranes were both cases important tools.

For extremely small pores the force of adhesion can be strong enough to hold the tip of the deforming polymer in the pore so tightly that the elongation reaches the point of break. In these cases, the hair ruptured and some polymer remains inside the pore. When this happens the template (in case of the here descript PC-template) needs to be replaced. For the pore diameters where all cylinders slide out of the pore before the elongation reached the point of break, the template can be used several times (Fig. 7).

The surface topography is undoubtedly of critical importance for the wetting behavior of the newly generated surfaces^31–35^. As seen in Table 1, the wetting properties of one and the same material could be tailored over a wide range of values by using different membranes, although its chemistry, i.e., its surface energy, remained *unchanged*. The water contact angle, CA, displayed by the original, unstructured HDPE film is *θ* = 94° ± 5°. Templating against the larger pores, *d* ≥ 5 μm decorates the HDPE surfaces with well-defined cylinder-shaped microstructures. The contact angle values of up to 160°obtained are, however, dominated by a large contact angle hysteresis. The strong pinning behavior results in water droplets, which are essentially immobile, even when surface is tilted very strongly (Fig. 2f). In contrast to this, demolding from membranes with *d* ≤ 5 µm generates surfaces with superhydrophobic properties (*θ* = 160° ± 10°; *θ*_roll-off_ < 10°). The presence of the extremely high AR polymer hairs (two orders of magnitude higher as compared to the structures obtained with pores of *d* = 10.0 μm) as well as their heterogeneous and entangled spatial arrangement makes it extremely difficult for the water droplets to stick to the surface (Fig. 2a–e). Furthermore, the re-entrant geometry of the characteristic bulge at their tips, formed during template removal^21^, could be an additional factor enhancing the water-repellent properties of such hairy surfaces^28,29^. First experiments on droplet impacts and the underwater behavior of trapped air layers indicate a surprisingly stable superhydrophobic state. Stable superhydrophobicity is obtained despite the fact, that these hairy structures are not further functionalized or coated with any other low surfaces energy material (e.g. fluorinated compounds).

## Conclusion

The filling of porous templates with molten polymer, followed by cooling and cold drawing of the polymer inside the pore, represents a method, which allows for a very simple generation of hairy surfaces consisting of dense lawns of highly elongated structures. Due to a delicate balance between the frictional force holding the frozen-in polymer in place in the pore and the yield force, surfaces that carry dense lawns of hairs with diameters of a few hundred nanometers and lengths reaching up to100 micrometers are formed within seconds. This is in so far an attractive as the process starts from a conventional polymer foil without the use of any specialized equipment. The AR of the structures realized this way have been up even over 200 and the hair densities of up to six hundred millions hairs per square centimeter. Such small and high aspect ratio polymer structures are not easily—if at all—attainable with conventional, well-established microreplication or lithographic techniques. The use of porous templates having different pore diameters allows the generation of the whole spectrum of hairy surfaces covered with short (‘crew cut’) or long and even curly hairs. The decoration of polymer surfaces with such high AR hairs allows the production of materials with very unusual surface properties without the need of any surface modification. While conventional surface modification commonly involves the deposition of non-polar molecules, usually fluorinated ones, the technique presented here can be used to transform even commodity polymers into superhydrophobic materials. The fabrication process described provides a convenient and low-cost way to create water-repellent polymer surfaces with unprecedented simplicity and speed.

## Methods

### Materials

HDPE films (thickness of ≈ 1 mm) were purchased from S-Polytec GmbH. Ion track-etched polycarbonate membranes with pore diameters of 0.6 μm, 1.0 μm, 3.0 μm, 5 µm and 10 μm were purchased from Whatman Nucleopore and those having 0.2 μm pore diameters were from Merck Millipore Ltd.

### Fabrication of HDPE nano- and microstructured substrates

The HDPE film (≈ 2 × 2 cm^2^) was allowed to heat up onto a hot plate at 170 °C. Subsequently, a polycarbonate porous membrane was placed on top of the polymer film, together with a small weight (≈ 100 g × cm^–2^) in order to ensure conformal contact between substrate and template. Note that the applied pressure does not have a large influence on the pore-assisted hair formation when weights are used in the range between 0 to 1 kg on top of the template during casting. The generation of hairs occurred even in the absence of any applied force, i.e., 0 N, suggesting that capillary rise of polymer melt within the PC pores takes place instantaneously. A too high pressure, however, leads to an overflow and formation of a polymer film on the other side of the membrane. The consequence of such an overflow is that the membrane cannot be separated from the substrate any more. A templating time between 15 – 600 s was sufficient to ensure the capillary rise of polymer melt into the micropores of the membrane. Template removal was carried out manually at room temperature.

### Chemical demolding

The degree of pore filling by the polymer was experimentally assessed by dissolving the polycarbonate membrane still in contact with the polymer substrate in dichloromethane overnight. By this method no tensile stress during demolding was applied to the newly formed nano- and microstructures. Accordingly, no drawing of the polymer occurred and the final length of the nano- and microstructures corresponds to the degree of pore filling with the polymer melt.

### Surface characterization

The various surface topographies were imaged by scanning electron microscopy (PhenomPro) with the accelerating voltage of 5 kV at a magnification of 2000 x. A higher magnification was not possible due to heat induced plastic deformations of the hairy structures at high intensities of the electron beam. Prior to imaging, all samples were sputtered with gold. The wetting behavior of the surfaces was assessed by measuring water contact angles with a sessile drop method using a Dataphysics OCA20 goniometer. The droplet volume employed was 10 µL (lower volumes could not be deposited onto the hairy surfaces due to their high water-repellent behavior). The hysteresis was defined as the difference between the advancing and receding contact angle in a dynamic measurement (ejection speed 0.2 µl/s). All measurements were carried out under ambient conditions. The final data were averaged by three measurements.
